# Family Support for Medical Nutritional Therapy and Dietary Intake among Japanese with Type 2 Diabetes (JDDM 56)

**DOI:** 10.3390/nu12092649

**Published:** 2020-08-31

**Authors:** Chika Horikawa, Mariko Hatta, Sakiko Yoshizawa Morikawa, Yasunaga Takeda, Mizuki Takeuchi, Kazuya Fujihara, Noriko Kato, Hiroki Yokoyama, Yoshio Kurihara, Koichi Iwasaki, Shiro Tanaka, Hiroshi Maegawa, Hirohito Sone

**Affiliations:** 1 Department of Health and Nutrition, University of Niigata Prefecture Faculty of Human Life Studies, 471 Ebigase, Higashi-ku, Niigata 950-8680, Japan; horikawa@unii.ac.jp; 2 Department of Hematology, Endocrinology, and Metabolism, Niigata University Faculty of Medicine, 1-757 Asahimachi-dori, Chuoh-ku, Niigata 951-8510, Japan; marichocolate.coffee02@gmail.com (M.H.); mr2.yac@gmail.com (Y.T.); y.mizuki3929@gmail.com (M.T.); kafujihara-dm@umin.ac.jp (K.F.); 3 Department of Food-Nutritional Sciences, Tokushima Bunri University Faculty of Human Life Sciences, 180 Hoji, Nishihama, Yamashiro-cho, Tokushima, Tokushima 770-8514, Japan; smorikawa@tks.bunri-u.ac.jp; 4 Kato Clinic of Internal Medicine, 201, 3-11-14 Takasago, Katsushika-ku, Tokyo 125-0054, Japan; norikokato.0131@icloud.com; 5 Jiyugaoka Medical Clinic, 6-4-3, Nishirokujominami, Obihiro, Hokkaido 080-0016, Japan; dryokoyama@yokoyamanaika.com; 6 Kurihara Clinic, 5-7-28, Atsubetsuchuosanjo, Sapporo Atsubetsu-ku, Hokkaido 004-0053, Japan; ykuri@yg7.so-net.ne.jp; 7 Iwasaki Naika Clinic, 1-30-13, Minamiiwakunimachi, Iwakuni, Yamaguchi 740-0034, Japan; kiwasaki@themis.ocn.ne.jp; 8 Department of Clinical Biostatistics, Graduate School of Medicine, Kyoto University, Yoshida-Konoe-cho, Sakyo-ku, Kyoto 606-8501, Japan; tanaka.shiro.8n@kyoto-u.ac.jp; 9 Department of Medicine, Division of Diabetology, Endocrinology and nephrology, Shiga University of Medical Science, Seta Tsukinowa-cho, Otsu, Sihga 520-2192, Japan; maegawa@belle.shiga-med.ac.jp

**Keywords:** type 2 diabetes mellitus, medical nutrition therapy, family support

## Abstract

The aim of this study was to investigate the association between habitual dietary intake for patients with diabetes and the content of family support for medical nutritional therapy (MNT). Analyzed were 289 Japanese with type 2 diabetes (men, 58.5%; mean age, 62.0 years; mean HbA1c, 53.4 mmol/mol) who completed the Food Frequency Questionnaire and Diabetes Family Behavior Checklist (DFBC). Relationships of mean values for food group intake to DFBC responses regarding MNT were examined using multivariate analysis of covariance. Positive response to “Praise for following diet” was associated with lower sweets intake (none: 60.1 g/day; ≥once monthly: 50.9 g/day, *p* = 0.038) and higher seasoning intake (none: 21.6 g/day, ≥once monthly: 24.1 g/day, *p* = 0.046). Energy intake was higher with positive responses to “Eat at the same time that you do” (none: 1636 kcal/day, ≥once monthly: 1818 kcal/day, *p* = 0.038). “Nags about not following diet” was associated with higher fish (none: 68.7 g/day, ≥once monthly: 78.7 g/day, *p* = 0.042) and salt intake (none: 8.3 g/day, ≥once monthly: 9.0 g/day, *p* = 0.014). Eating foods not part of the diabetic diet (none: 218.4 g/day, ≥once monthly: 246.9 g/day, *p* = 0.014) resulted in a higher vegetable intake. In females, significant differences in relationships in the overall analysis were reversed. Our results clarified relationships between types of family support of patients with type 2 diabetes and their dietary intake and the importance of sex differences for more effective MNT.

## 1. Introduction

Self-care activities in the treatment of diabetes are crucial for improving glycemic control [1,2], reducing the risk of diabetes-related complications [2,3], and improving the prognosis [4]. However, performing ongoing self-care activities is sometimes difficult and adherence to medical nutrition therapy (MNT) has been shown to be poorer than adherence to medications [5,6], though MNT is an essential component of diabetes treatment. 

Psychosocial care plays an important role in the practice of good self-care activities and has been associated with reducing stress, promoting positive emotions and behaviors [7,8], improving adherence to recommended treatment regimens, improving glycemic control [8], and preventing the aggravation of the disease [9]. In psychosocial care, family support is the most easily accessible [10]. Our previous meta-analysis of interventional studies (follow-up period: 3.5–30 months) showed that HbA1c in diabetic patients was significantly lower when family members were involved than were not involved in family-oriented diabetes programs [11].

After completion of family-oriented programs for patients with diabetes, it was reported that self-efficacy in self-care activities improved, including adherence to MNT and adopting preferred dietary habits and intakes, such as more frequently selecting fat-reduced cooking methods such as baking or grilling, avoiding foods containing high amounts of fat and sugar, reducing fat intake, and increasing intakes of vegetables and fruits [12,13]. HbA1c levels were also shown to be lower in patients with type 2 diabetes who had family support for MNT, who shared education sessions for MNT, engaged in patient-family member communication about MNT, and received assistance with food preparation and grocery shopping [13,14,15].

Types of family support of diabetes patients have various and important effects on maintaining lifestyle changes and diabetes self-management. For example, it was reported that obstructive family behaviors with regard to patients’ self-care efforts in diabetes care are barriers to effective self-efficacy and self-management adherence [13,16,17]. With respect to MNT, pressure and obstruction from family members regarding dietary matters were associated with lower dietary self-care adherence, higher diabetes-specific distress, and worsening blood glucose control among patients with type 2 diabetes [17,18,19]. However, evidence is sparse on the relationship between average dietary intake by food group and nutrient level and the content of family support regarding MNT in patients with diabetes. Without considering this relationship, it is difficult to determine whether such patients are receiving appropriate family support and to formulate effective family-oriented programs for MNT. Therefore, we investigated whether family support for MNT in Japanese outpatients with type 2 diabetes was related to average daily food and nutritional intake considering types of family support for MNT and patients’ gender.

## 2. Materials and Methods

### 2.1. Study Population

Practicing diabetologists in eight diabetes clinics that were part of the Japan Diabetes Clinical Data Management Study Group (JDDM) participated in this cross-sectional study. Details of the JDDM have been described elsewhere [20,21]. Potential participants were 354 patients with type 2 diabetes aged ≥20 years who underwent medical treatment in outpatient clinics from November 2015 to September 2018. The patients were requested to complete the Japanese version of the Diabetes Family Behavior Checklist (DFBC) [22,23] and the Food Frequency Questionnaire based on food groups (FFQg) [24]. Of these, 289 completed the questionnaires (collection rate, 81.6%), and their data were analyzed. The protocol for the study, which is in accordance with the Declaration of Helsinki and the Ethical Guidelines for Clinical/Epidemiological Studies of the Japanese Ministry of Health Labor and Welfare, received ethical approval from the institutional review boards of all participating institutes and the ethics committee of the Niigata University Faculty of Medicine, Japan (No. 2317). Written informed consent was obtained from all participants.

### 2.2. Family Support for MNT

Family support for MNT was measured by the Japanese version of the DFBC. This instrument showed validity and reliability for the quick assessment of self-managed treatment behavior of Japanese type 2 diabetes patients [23]. It consists of ‘‘positive feedback’’ and ‘‘negative feedback’’ questions on five aspects of diabetes treatment: daily living, drug therapy, blood glucose measurement, exercise, and MNT. Patients answered questions by selecting ‘‘None,’’ ‘‘Once a month,’’ ‘‘Once a week,’’ ‘‘Several times a week,’’ or ‘‘At least once a day.’’ The present study used diet regimen-specific subscales because previous studies using regimen-specific subscales such as diet and exercise reported that subscales were stronger indicators of their respective areas of regimen adherence than either overall positive or negative summary scores [12,25,26]. The diet regimen-specific subscale was used, which is composed of two positive and two negative items to assess family support specific to MNT. To elicit positive feedback, items were how often a particular family member would “praise you for following your diet” and “eat at the same time that you do.” Negative feedback was determined by items on how often a family member “nagged you about not following your diet” and how often did family members “eat foods that are not part of your diabetes diet.”

### 2.3. Dietary Assessment

Nutrition and food intakes were assessed by the FFQg. The FFQg is composed of items on 29 food groups and 10 kinds of cookery and elicits information on the average intake per week of each food or food group in commonly used units or portion sizes. After participants completed the questionnaire, a dietician reviewed the completed questionnaire with the participant. The FFQg was externally validated by comparison with weighed dietary records for seven continuous days of 66 individuals aged 19–60 years [24].

The correlation coefficients between the FFQg and 7-d dietary records for energy, protein, fat, and carbohydrate intakes were 0.47, 0.42, 0.39, and 0.49, respectively, and intakes of rice, bread, meat, fish, milk, dairy products, green-yellow vegetables, other vegetables, and fruits were 0.66, 0.76, 0.27, 0.27, 0.72, 0.58, 0.46, 0.53, and 0.64, respectively. Intakes of 26 of the 31 nutrients and 22 of the 29 food groups were not significantly different between the two methods by paired t tests. We used standardized software for population-based surveys and nutrition counseling in Japan (EIYO-KUN, manufactured at the site of the Shikoku University Nutrition Database) based on the Standard Tables of Food Composition in Japan [27] edited by the Japanese Ministry of Education, Culture, Sports, Science, and Technology to calculate nutrient and food intakes.

### 2.4. Other Assessments

HbA1c was measured in each clinic by high performance liquid chromatography. HbA1c assays were standardized by the Lab Test Committee of the Japan Diabetes Society (JDS) [28]. HbA1c values were converted from JDS values into National Glycohemoglobin Standardization Program (NGSP) equivalent values. NGSP equivalent values were calculated using the following formula: NGSP equivalent value (%) = JDS value (%) + 0.416.

Data on participants’ age, sex, duration of diabetes, height, weight, body mass index (BMI), and use of oral hypoglycemic agents (OHA) and insulin were obtained by medical records from each diabetes clinic enrolled in this study. Participants were also asked whether he or she lived with a family member and who provided the strongest family support.

### 2.5. Statistical Analysis

Categorical variables were expressed as numerals and percentages and were compared with Fisher’s exact test or the χ^2^ test. Continuous variables were expressed as mean ± SD and were compared using the t test for comparisons in each group. Multiple regression analysis was used to assess the relation of energy, nutrients, and food intakes with the answer to the DFBC according to presence or absence of family support for MNT after accounting for potential confounders. We divided the answers of the diet regimen-specific subscale between none and greater or equal to once a month to investigate presence or absence of each type of family support for MNT. Energy intake was adjusted for age, sex, duration of diabetes, BMI, HbA1c, using OHA, and using insulin. Nutrients and food group intakes were adjusted for age, sex, duration of diabetes, BMI, HbA1c, using OHA, using insulin, and energy intake per day. All analyses were performed by SPSS (version 23.0, IBM, New York, NY, USA), and statistical significance was considered for *p* < 0.05.

## 3. Results

Table 1 shows the characteristics of the 289 type 2 diabetes patients. Participants’ mean age was 62.0 years, mean duration of diabetes was 12.2 years, and 58.5% were men. Their mean HbA1c value was 53.4 mmol/mol and mean BMI was 25.8 kg/m^2^. An OHA and insulin were used by 46.4% and 14.2% of participants, respectively. There were no significant differences in age, diabetes duration, HbA1c level, BMI, and usage of an OHA and insulin between sexes.

As to family support for diabetes care, among male patients, 87.6% were mainly supported by wives, while 65.8% of female patients were mainly supported by husbands and 16.6% obtained the strongest family support from their children (*p* < 0.001). Most patients lived with their family regardless of sex (men, 93.5%; women, 92.5%, *p* = 0.816). As for positive feedback on the DFBC scale for MNT, no significant difference was observed between participants’ sex, and the rates of responses of ≥once a month to “praise you for following your diet” and “eat at the same time that you do” were 46.4% and 82.7%, respectively. Regarding negative feedback, men had a significantly higher rate of responses of ≥once a month to “nag you about not following your diet” than women (55.0% and 29.2%, *p* < 0.001), but there was no significant difference in the responses to ≥once a month to “eat foods that are not part of your diabetes diet” (41.4% and 50.0%, *p* = 0.339).

The associations between dietary intake and responses to the DFBC scale for MNT are shown in Table 2. After adjusting for confounders, protein intake and proportion of protein as the percentage of energy supply were significantly greater in patients who received positive family support (i.e., praise for following diet) compared with those who did not. However, the differences were small clinically. Lower intake of sweets and higher intake of seasonings were observed in those who indicated praise for the following food than in those indicating “none” (sweets: none 60.1 g/day, ≥once a month 50.9 g/day, *p* = 0.038; and seasonings: none 21.6 g/day, ≥once a month 24.1 g/day, *p* = 0.046). There were no significant associations between the responses to this item and other nutrient and food group intakes. Energy intake was significantly higher among those with a positive response to “eat at the same time that you do” than with a negative response (none 1636 kcal/day, ≥1818 kcal/day, *p* = 0.038). There were no significant differences in nutrient and food group intakes according to responses to this item. In relation to negative family support, selecting “nag about not following diet” was significantly associated with higher fish intake (none 68.7 g/day; ≥once a month 78.7 g/day, *p* = 0.042) and high salt and sugar intakes (salt, none 8.3 g/day; ≥once a month 9.0 g/day, *p* = 0.014; sugar, none 6.5 g/day; ≥ once a month 8.3 g/day, *p* = 0.004). However, the difference in sugar intake was small in the daily diet. Those indicating “eat foods not part of the diabetes diet” had a higher fiber intake (none 12.5 g/day, ≥once a month 13.5 g/day, *p* = 0.008) and total vegetable intake (including “other vegetables” category) than those who answered “none” (total vegetables: none: 218.4 g/day, ≥once a month 246.9 g/day, *p* = 0.014, other vegetables intake: none 140.0 g/day, ≥once a month 160.1 g/day, *p* = 0.004).

Table 3 shows results of the subgroup analysis of men with type 2 diabetes according to dietary intake and responses to the DFBC scale for MNT. There were no notable differences in intake of nutrients and food groups according to baseline characteristics regardless of the response to “praise for following your diet.” There was a significant difference only in energy intake among men who responded positively to “eat at the same time that you do” (none 1602 kcal/day, ≥once a month: 1910 kcal/day, *p* = 0.038). As for responses to “nag you about not following your diet,” significantly higher fish, seaweed, and salt intake and lower intake of beverages except for alcohol were shown (fish: none 66.6 g/day, ≥once a month 80.7 g/day, *p* = 0.034; seaweed: none 4.0 g/day, ≥once a month 5.3 g/day, *p* = 0.027; salt: none 8.3 g/day, ≥once a month 9.2 g/day, *p* = 0.032; beverages except alcohol: none 82.6 g/day, ≥once a month or more 48.8 g/day, *p* = 0.004). In those indicating “eat foods not part of your diabetes diet,” the intakes of fiber and milk/dairy products were significantly higher (fiber: none 11.8 g/day, ≥once a month 12.8 g/day, *p* = 0.040; milk/dairy products: none 71.7 g/day, ≥once a month 96.2 g/day, *p* = 0.015).

With regard to women with type 2 diabetes (Table 4), the relationships with dietary intake and the responses to “praise for following your diet,” “eat at the same time that you do,” and “eat foods that are not part of your diabetes diet” were not significant. A significant difference was only shown when the female patients selected “nag about not following your diet.” This expression of negative family support was only associated with high sugar intake (none 7.4 g/day, ≥once a month 9.6 g/day, *p* = 0.015), but this difference was small as part of the dietary intake.

## 4. Discussion

The current study determined the association between habitual dietary intake by food group and nutrient levels among Japanese with type 2 diabetes and types of daily family support for MNT using DFBC. 

The present results suggest that family members regularly discussing MNT with patients with type 2 diabetes by such means as “praise for following your diet” or “nag about not following your diet” was related to patients’ intake of desirable food groups in comparison with other cases in which such conversations did not take place, though there was no significant difference in energy intake. Patients with type 2 diabetes need family support and assistance in self-management practices through help with strategic planning, goal setting, and problem-solving [29]. Patient-family relationships have been shown to improve adherence to general dietary recommendations and motivation for diabetes treatment [13,19,29]. Although it is considered important in MNT for diabetes to provide and put into practice specific indications, increases in patients’ self-efficacy including a positive attitude toward treatment can arise from positive support such as praise and encouragement [30,31]. Our results showed that those responding positively to “nag about not following your diet” had a higher intake of fish and shellfish than those who responded “none.” To maintain this result over the long term, it would be necessary for family members to encourage patients to increase fish and shellfish intake while “praising the patient for following their diet.”

In addition, the intake of salt was significantly higher in study participants selecting the response indicating that they experienced nagging about not following their diet. The amount of salt intake is difficult to grasp visually as salt is found in seasonings and processed foods [32,33]. It was reported that seasonings contributed to about 60% of the salt intake in Japanese people [34]. In MNT for type 2 diabetes, there is a need to educate patients and their family members on how to reduce salt intake such as the characteristics of food with a high-salt content, how to read nutritional information on labels, and how to reduce salt in cooking [34,35,36]. This information is important when educating patients on adequate intake of energy and certain food groups.

As for “eat at the same time that you do,” patients who indicated that this behavior occurred once a month or more had a higher energy intake compared to those with a response of “none,” but no significant difference was seen in intakes of food groups and nutrients. This indicates that family members of patients with type 2 diabetes may not have a good understanding of what food groups and how much food intake are effective for MNT when eating with the patient. An international joint research project reported that a low percentage of family members of people with diabetes know how to help persons with their diabetes (37.1%) and participate in diabetes educational programs (23.1%), although most of those who did participate found such programs helpful (72.1%) [37]. A family member should be present at the time of the MNT education to share information on dietary intake in diabetes care and closely support a patient’s MNT. Such participation will enhance the patient’s self-efficacy, including that related to MNT, encourage a positive attitude toward treatment, and increase the consumption of healthy foods such as vegetables and fruit [12,13].

Patients indicating that they "eat foods that are not part of their diabetes diet" had higher dietary fiber and vegetable intake than those who did not. High dietary fiber intake including that of vegetables is also important for family members without diabetes because MNT for diabetes is also helpful in the prevention of many lifestyle-related diseases, and not only diabetes [38,39,40]. By having both patients with type 2 diabetes and their family members consuming recommended daily values for vegetables and dietary fiber (fiber intake: ≥14 g/1000kcal by the American Diabetes Association [41] and ≥20 g/day by the Japan Diabetes Society [42]), the whole family would benefit by avoiding lifestyle-related diseases and have a higher quality of life.

When subgroup analysis by sex was performed, the results for male patients roughly followed the same pattern for significant differences as in the overall study population. Some additional relationships, such as higher fiber intake, higher seaweed intake, and fewer preferred beverages other than alcohol were shown for negative feedback such as “being nagged about not following diet” and “eating foods that are not part of the diabetes diet.” On the other hand, for female patients, significant differences were attenuated in the areas where significant differences were found in the overall study population. This suggests that familial dietary support is associated with preferred habitual dietary intake, but this tendency was especially strong for male patients and a reverse association was found in female patients.

The reason why support for MNT by family members was significantly associated with good habitual dietary intake in males but not in females may be because the attributes of those providing the support were different. According to Table 1, most male patients (87.6%) received support from their wives while 65.8% of females received support from husbands and 16.6% from children. 

In daily eating, food and nutritional knowledge regarding the composition of healthy meals greatly affects the content of the meals and varies according to sex. Women tend to desire knowledge about food and nutrients to a greater degree than men [43,44]. This knowledge can result in eating a diet rich in fruits and vegetables and low in fats [43,45]. Furthermore, it has been reported that 76% of women are in charge of shopping for groceries, and 91% of women are mainly involved in preparing meals in Japanese families [46]. In addition, it was reported that knowledge of food and nutrients as well as practical skills in food selection and preparation are usually at a low level in young age groups [44,45].

In summary, male patients are most likely to receive support regarding knowledge of food and nutrients from their wives who have knowledge of nutrition and practical skills in food preparation and female patients receive support from husbands and children who have less knowledge of food. Also, most female patients are in charge of preparing daily meals. Hence, it can be expected that female patients are likely to work on MNT on their own, and that family support would not lead to dietary improvements. In order to prevent disparities in the support situation due to sex differences, care should be taken to ensure that family support for female patients is effectively linked to MNT by involving family members, so that they may acquire basic food knowledge and cooking skills.

Limitations of this study must be considered. First, the potential for bias, such as measurement errors in dietary assessments, confounding factors, and informative censoring, cannot be ruled out entirely although this study is adjusted for age, sex, duration of diabetes, BMI, HbA1c, using OHA, and using insulin as confounders for dietary intake using multiple regression analysis. Second, as this is a cross-sectional study, cause and effect relationships could not be concluded. Further cohort studies and randomized trials are needed for clarify whether family support encouraging MNT would affect habitual dietary intake in clinical practice. Third, although 81.6% of patients with type 2 diabetes completed our survey, the potential effect of a partial non-response bias should be addressed. It is known that people who participate in research studies tend to have health awareness and be well motivated compared with those who do not [47,48]. Finally, our results may not be applicable to populations with different lifestyles or genetic factors. For example, the Japanese patients with type 2 diabetes were shown to consume a high-carbohydrate low-fat diet compared with Western patients with diabetes [49], and their mean BMI was low compared to persons from Western countries [50]. It is also known that racial/ethnic variations differ among family household compositions and family dynamics [17]. Considering racial/ethnic-specific characteristics and large intercultural differences is important in exploring effective MNT. Further research on this topic is needed. 

## 5. Conclusions

In conclusion, we clarified in detail the relationships between the type of family support received by patients with type 2 diabetes in daily life and their dietary intake by food group and nutrient level. We showed that support required for medical treatment, which involves not only negative feedback but also positive feedback among family support, can be linked to desirable dietary intake for diabetes care, and that it is necessary to pay particular attention to family support for women. Further longitudinal studies or interventional studies are needed for tracking the dietary intake of patients with type 2 diabetes, and exploring family support in consideration of sex difference is important for the more effective practice of MNT.

## Figures and Tables

**Table 1:** Characteristics of study participants.

	Overall (*N* = 289)				Men (*N* = 169)				Women (*N* = 120)					
	N	Mean	±	SD	N	Mean	±	SD	N	Mean	±	SD	p	
Age (year)	289	62.0	±	11.3	169	61.9	±	10.8	120	62.2	±	12.1	0.837	†
Men, %	169	58.5	%		169	100.0	%		0	0.0	%		-	
Living with family, %	268	92.7	%		157	93.5	%		111	92.5	%		0.816	‡
Supported mainly by husband, %	78	27.0	%		0	0.0	%		79	65.8	%		<0.001	§
Supported mainly by wife, %	144	49.8	%		148	87.6	%		0	0.0	%			
Supported mainly by their children, %	29	17.2	%		1	0.6	%		28	16.6	%			
Supported mainly by their parents, %	26	15.4	%		17	10.1	%		9	5.3	%			
Supported mainly by others not above, %	12	7.1	%		3	1.8	%		4	2.4	%			
Duration of diabetes (year)	289	12.2	±	7.3	169	12.4	±	0.6	120	11.9	±	0.7	0.547	†
Height (cm)	289	162.1	±	8.6	169	167.2	±	0.5	120	154.9	±	0.5	<0.001	†
Weight (kg)	289	67.9	±	15.7	169	71.4	±	1.1	120	63.0	±	1.4	<0.001	†
BMI (kg/cm^2^)	289	25.8	±	5.0	169	25.5	±	0.3	120	26.1	±	0.5	0.380	†
HbA1c (mmol/mol)	289	53.4	±	6.4	169	52.5	±	5.4	120	54.7	±	7.5	0.006	†
Using OHA, %	134	46.4	%		83	49.1	%		51	42.5	%		0.283	‡
Using insulin, %	41	14.2	%		20	11.8	%		21	17.5	%		0.231	‡
DFBC scale for MNT														
Positive feedback														
- Praise you for following your diet														
None	155	53.6	%		79	46.7	%		76	63.3	%		0.075	§
Once a month	45	15.6	%		31	18.3	%		14	11.7	%			
Once a week	34	11.8	%		21	12.4	%		13	10.8	%			
Several times a week	44	15.2	%		31	18.3	%		13	10.8	%			
At least once a day	11	3.8	%		7	4.1	%		4	3.3	%			
- Eat at the same time that you do														
None	50	17.3	%		30	17.8	%		20	16.7	%		0.718	§
Once a month	7	2.4	%		5	3.0	%		2	1.7	%			
Once a week	6	2.1	%		2	1.2	%		4	3.3	%			
Several times a week	41	14.2	%		24	14.2	%		17	14.2	%			
At least once a day	185	64.0	%		108	63.9	%		77	64.2	%			
Negative feedback														
- Nag you about following your diet														
None	161	55.7	%		76	45.0	%		85	70.8	%		<0.001	§
Once a month	61	21.1	%		44	26.0	%		17	14.2	%			
Once a week	32	11.1	%		25	14.8	%		7	5.8	%			
Several times a week	26	9.0	%		20	11.8	%		6	5.0	%			
At least once a day	9	3.1	%		4	2.4	%		5	4.2	%			
- Eat foods that are not part of your diabetic diet														
None	159	55	%		99	58.6	%		60	50.0	%		0.339	§
Once a month	21	7.3	%		14	8.3	%		7	5.8	%			
Once a week	23	8.0	%		13	7.7	%		10	8.3	%			
Several times a week	41	14.2	%		22	13.0	%		19	15.8	%			
At least once a day	45	15.6	%		21	12.4	%		24	20.0	%			

†. t test, ‡. Fisher’s exact test, §. Chi-squared test. BMI, body mass index; DFBC, Diabetes Family Behavior Checklist; MNT, medical nutrition therapy; OHA, oral hypoglycemic agent.

**Table 2:** Dietary intake and response to the Diabetes Family Behavior Checklist (DFBC) scale for MNT in patients with type 2 diabetes (*N* = 289).

																			Eat Foods that are not Part of					
	Praise You for Following Your Diet						Eat at the Same Time that You do						Nag You about Following Your diet						Your Diabetic Diet					
	None		≥ Once a Month				None		≥ Once a Month				None		≥ Once a Month				None		≥ Once a Month			
	Mean	[95%CI]	Mean	[95%CI]	*p*		Mean	[95%CI]	Mean	[95%CI]	*p*		Mean	[95%CI]	Mean	[95%CI]	*p*		Mean	[95%CI]	Mean	[95%CI]	*p*	
No. participants	155		134				50		239				161		128				159		130			
Energy (kcal)	1792	[1712–1873]	1780	[1693–1867]	0.839		1636	[1498–1774]	1818	[1755–1881]	0.019	*	1745	[1666–1823]	1839	[1751–1928]	0.124		1769	[1690–1848]	1808	[1721–1896]	0.515	
Protein (g)	64.0	[62.5–65.5]	66.4	[64.8–68]	0.038	*	64.0	[61.3–66.6]	65.3	[64.1–66.5]	0.352		64.6	[63.1–66.1]	65.7	[64.0–67.4]	0.364		64.9	[63.5–66.4]	65.3	[63.6–66.9]	0.772	
Protein (% Energy)	14.3	[14–14.6]	14.8	[14.5–15.2]	0.027	*	14.4	[13.8–14.9]	14.6	[14.3–14.8]	0.499		14.5	[14.2–14.8]	14.6	[14.3–15.0]	0.510		14.5	[14.1–14.8]	14.7	[14.3–15]	0.409	
Fat (g)	57.8	[55.9–59.7]	58.1	[56–60.1]	0.836		57.7	[54.5–61]	58.0	[56.5–59.4]	0.906		57.6	[55.8–59.5]	58.3	[56.2–60.3]	0.658		58.0	[56.2–59.9]	57.8	[55.7–59.8]	0.866	
Fat (% Energy)	28.6	[27.7–29.5]	29.3	[28.3–30.2]	0.290		28.7	[27.2–30.2]	29.0	[28.3–29.6]	0.772		28.8	[27.9–29.6]	29.1	[28.1–30.0]	0.658		28.8	[28.0–29.7]	29.0	[28.1–30]	0.785	
Carbohydrate (g)	238.3	[232.8–243.7]	236.0	[230.2–241.9]	0.590		238.5	[229.0–248.0]	237.0	[232.7–241.3]	0.769		238.8	[233.5–244.2]	235.2	[229.2–241.2]	0.385		236.0	[230.7–241.3]	238.7	[232.8–244.6]	0.511	
Carbohydrate (% Energy)	57.1	[56–58.2]	55.9	[54.7–57.0]	0.128		56.9	[55.0–58.8]	56.5	[55.6–57.3]	0.663		56.7	[55.7–57.8]	56.3	[55.1–57.5]	0.584		56.7	[55.7–57.8]	56.3	[55.2–57.5]	0.628	
Fiber (g)	12.8	[12.3–13.3]	13.1	[12.5–13.6]	0.484		13.3	[12.4–14.2]	12.8	[12.4–13.2]	0.368		12.9	[12.4–13.4]	12.9	[12.3–13.4]	0.873		12.5	[12–12.9]	13.5	[12.9–14.0]	0.008	*
Salt (g)	8.3	[8.0–8.7]	8.9	[8.5–9.3]	0.053		8.2	[7.5–8.8]	8.7	[8.4–9.0]	0.185		8.3	[7.9–8.6]	9.0	[8.6–9.4]	0.014	*	8.5	[8.1–8.9]	8.7	[8.3–9.1]	0.462	
Potassium (mg)	2200	[2129–2271]	2244	[2168–2321]	0.408		2237	[2113–2360]	2217	[2161–2273]	0.776		2219	[2149–2288]	2223	[2145–2301]	0.937		2181	[2112–2250]	2269	[2193–2346]	0.097	
Grain (g)	367.9	[349.0–386.8]	372.1	[351.7–392.5]	0.768		358.1	[325.2–391.1]	372.3	[357.3–387.2]	0.443		372.7	[354.2–391.2]	366.2	[345.4–387.1]	0.657		368.3	[349.8–386.9]	371.7	[351.1–392.3]	0.813	
Potato (g)	27.8	[23.5–32]	31.6	[27.0–36.2]	0.236		30.2	[22.8–37.7]	29.4	[26.0–32.8]	0.842		27.8	[23.7–32]	31.7	[27.0–36.4]	0.237		27.9	[23.7–32.0]	31.6	[27.0–36.2]	0.246	
Vegetables (g)	228.4	[210.8–246]	234.4	[215.5–253.4]	0.652		235.2	[204.5–265.9]	230.4	[216.4–244.3]	0.779		234.9	[217.6–252.1]	226.6	[207.1–246]	0.539		218.4	[201.3–235.5]	246.9	[227.9–265.8]	0.031	*
Green-yellow vegetables (g)	82.0	[75.0–89.1]	82.3	[74.7–89.9]	0.959		82.4	[70.1–94.7]	82.1	[76.5–87.7]	0.965		83.9	[77–90.9]	79.9	[72.1–87.7]	0.455		78.4	[71.5–85.3]	86.8	[79.1–94.4]	0.114	
Other vegetables (g)	146.4	[134.7–158.1]	152.2	[139.5–164.8]	0.517		152.8	[132.4–173.2]	148.3	[139–157.5]	0.693		150.9	[139.5–162.4]	146.7	[133.8–159.6]	0.637		140.0	[128.7–151.4]	160.1	[147.5–172.7]	0.023	*
Seaweeds (g)	4.9	[4.3–5.5]	5.5	[4.9–6.2]	0.174		5.3	[4.2–6.4]	5.2	[4.7–5.6]	0.791		4.9	[4.3–5.5]	5.6	[4.9–6.3]	0.115		4.8	[4.2–5.4]	5.6	[4.9–6.3]	0.108	
Soybeans/Soy products (g)	61.0	[55.0–67.0]	65.9	[59.4–72.4]	0.285		68.4	[57.9–78.9]	62.2	[57.4–67.0]	0.295		64.3	[58.4–70.2]	62.0	[55.3–68.7]	0.621		63.0	[57–68.9]	63.7	[57.1–70.2]	0.879	
Fish/Processed fish (g)	69.3	[62.9–75.7]	77.5	[70.7–84.4]	0.091		68.2	[57–79.3]	74.1	[69.1–79.2]	0.338		68.7	[62.5–74.9]	78.7	[71.6–85.7]	0.042	*	73.7	[67.5–80.0]	72.3	[65.4–79.3]	0.770	
Meat/Processed meat (g)	73.5	[66.7–80.4]	70.0	[62.6–77.4]	0.497		67.2	[55.2–79.1]	72.9	[67.5–78.3]	0.394		70.8	[64.1–77.5]	73.2	[65.6–80.8]	0.650		71.3	[64.6–78.0]	72.6	[65.2–80.1]	0.790	
Eggs (g)	26.9	[24.3–29.6]	28.5	[25.7–31.4]	0.437		29.6	[25–34.2]	27.3	[25.2–29.4]	0.367		28.3	[25.6–30.9]	27.0	[24.0–29.9]	0.523		27.6	[25–30.2]	27.7	[24.8–30.6]	0.970	
Milk/Dairy products (g)	121.7	[107.7–135.7]	132.9	[117.9–148.0]	0.292		122.4	[98.0–146.9]	127.8	[116.8–138.9]	0.692		129.6	[115.9–143.4]	123.5	[108.0–139.0]	0.568		132.8	[119.1–146.5]	119.7	[104.5–134.9]	0.215	
Fruits (g)	91.1	[81.4–100.7]	86.0	[75.5–96.4]	0.489		93.9	[76.9–110.8]	87.6	[79.9–95.3]	0.510		83.2	[73.8–92.7]	95.6	[84.9–106.2]	0.096		84.8	[75.3–94.3]	93.5	[82.9–104]	0.237	
Sweets (g)	60.1	[54.3–66.0]	50.9	[44.6–57.1]	0.038	*	62.8	[52.6–73.0]	54.4	[49.7–59]	0.142		57.2	[51.4–63]	54.1	[47.6–60.6]	0.491		57.9	[52.2–63.7]	53.3	[46.9–59.7]	0.298	
Alcoholic beverages (g)	76.4	[58.8–94.0]	68.4	[49.4–87.4]	0.552		78.4	[47.6–109.2]	71.5	[57.5–85.5]	0.691		68.1	[50.8–85.4]	78.5	[59.0–98.0]	0.440		79.7	[62.4–97]	64.1	[45–83.3]	0.242	
Other beverages (g)	51.9	[37.6–66.2]	44.4	[28.9–59.8]	0.489		53.3	[28.3–78.3]	47.4	[36.1–58.7]	0.675		57.3	[43.3–71.2]	37.3	[21.5–53]	0.069		47.4	[33.4–61.5]	49.7	[34.1–65.2]	0.836	
Sugar (g)	7.1	[6.3–7.8]	7.6	[6.7–8.4]	0.393		7.0	[5.6–8.3]	7.4	[6.7–8.0]	0.614		6.5	[5.8–7.3]	8.3	[7.4–9.1]	0.004	*	7.0	[6.2–7.7]	7.7	[6.9–8.6]	0.189	
Nuts (g)	2.8	[2.1–3.5]	2.7	[2.0–3.5]	0.902		2.3	[1.1–3.4]	2.9	[2.4–3.4]	0.331		2.8	[2.1–3.4]	2.7	[2.0–3.5]	0.930		2.6	[2.0–3.3]	3.0	[2.2–3.7]	0.477	
Oil (g)	11.3	[10.2–12.4]	12.0	[10.8–13.2]	0.380		10.3	[8.4–12.2]	11.9	[11–12.8]	0.133		11.1	[10.0–12.2]	12.3	[11.1–13.5]	0.176		11.7	[10.6–12.7]	11.6	[10.4–12.8]	0.963	
Seasonings (g)	21.6	[20–23.3]	24.1	[22.4–25.9]	0.046	*	20.8	[17.9–23.7]	23.2	[21.9–24.5]	0.138		22.1	[20.4–23.7]	23.7	[21.9–25.5]	0.196		23.0	[21.4–24.6]	22.5	[20.7–24.3]	0.698	

* *p* < 0.05. Energy intake was adjusted for age, sex, duration of diabetes, BMI, HbA1c, using OHA, and using insulin. Nutrients and food group intakes were adjusted for age, sex, duration of diabetes, BMI, HbA1c, using OHA, using insulin, and energy intake per day. BMI, body mass index; OHA, oral hypoglycemic agent.

**Table 3:** Dietary intake and response to the Diabetes Family Behavior Checklist scale for medical nutritional therapy in men with type 2 diabetes (*N* = 169).

																			Eat Foods that are not Part of					
	Praise You for Following Your Diet						Eat at the Same Time that You Do						Nag You about Following Your Diet						Your Diabetic Diet					
	None		≥ Once a Month				None		≥ Once a Month				None		≥ Once a Month				None		≥ Once a Month			
	Mean	[95%CI]	Mean	[95%CI]	*p*		Mean	[95%CI]	Mean	[95%CI]	*p*		Mean	[95%CI]	Mean	[95%CI]	*p*		Mean	[95%CI]	Mean	[95%CI]	*p*	
No. participants	79		90				30		139				76		93				99		70			
Energy (kcal)	1849	[1725–1973]	1861	[1745–1977]	0.888		1602	[1407–1797]	1910	[1820–2000]	0.005	*	1821	[1696–1946]	1884	[1771–1997]	0.466		1824	[1712–1936]	1900	[1766–2034]	0.400	
Protein (g)	64.5	[62.3–66.8]	67.3	[65.2–69.5]	0.080		63.3	[59.5–67.0]	66.6	[64.9–68.3]	0.109		65.0	[62.7–67.3]	66.9	[64.8–68.9]	0.246		65.9	[63.9–68]	66.2	[63.7–68.7]	0.875	
Protein (% Energy)	13.9	[13.5–14.4]	14.4	[14–14.9]	0.116		13.8	[13.0–14.5]	14.3	[14.0–14.6]	0.205		14.1	[13.6–14.5]	14.3	[13.9–14.7]	0.409		14.2	[13.8–14.6]	14.2	[13.7–14.7]	0.861	
Fat (g)	58.9	[56–61.8]	58.6	[55.9–61.3]	0.891		58.4	[53.6–63.1]	58.8	[56.6–61.0]	0.880		58.3	[55.4–61.3]	59.0	[56.4–61.7]	0.735		58.9	[56.3–61.5]	58.5	[55.4–61.6]	0.862	
Fat (% Energy)	28.2	[26.9–29.5]	28.4	[27.2–29.6]	0.788		28.5	[26.4–30.6]	28.3	[27.3–29.2]	0.862		28.2	[26.9–29.5]	28.4	[27.2–29.6]	0.852		28.4	[27.2–29.5]	28.2	[26.8–29.6]	0.851	
Carbohydrate (g)	246.6	[238.1–255.2]	245.6	[237.6–253.6]	0.866		248.5	[234.5–262.5]	245.6	[239.2–252.0]	0.711		248.7	[240.1–257.3]	243.9	[236.2–251.7]	0.421		244.2	[236.5–251.9]	248.7	[239.5–257.9]	0.476	
Carbohydrate (% Energy)	57.9	[56.3–59.4]	57.1	[55.7–58.6]	0.496		57.8	[55.2–60.3]	57.4	[56.3–58.6]	0.819		57.7	[56.2–59.3]	57.3	[55.9–58.7]	0.694		57.4	[56–58.8]	57.6	[55.9–59.2]	0.924	
Fiber (g)	12.0	[11.4–12.7]	12.4	[11.8–13.0]	0.420		12.5	[11.4–13.6]	12.2	[11.7–12.7]	0.614		12.1	[11.5–12.8]	12.3	[11.7–12.9]	0.741		11.8	[11.2–12.4]	12.8	[12.1–13.5]	0.040	*
Salt (g)	8.4	[7.8–9.0]	9.2	[8.6–9.7]	0.066		8.4	[7.4–9.3]	8.9	[8.5–9.4]	0.294		8.3	[7.8–8.9]	9.2	[8.7–9.7]	0.032	*	8.7	[8.2–9.2]	9.0	[8.3–9.6]	0.594	
Potassium (mg)	2147	[2053–2241]	2176	[2088–2264]	0.663		2161	[2006–2316]	2163	[2092–2233]	0.988		2159	[2064–2255]	2165	[2079–2251]	0.932		2126	[2042–2211]	2213	[2112–2315]	0.208	
Grain (g)	389.6	[360.7–418.4]	399.9	[373.0–426.9]	0.609		385.4	[337.9–432.9]	397.2	[375.6–418.8]	0.660		398.8	[369.6–428.0]	392.1	[365.7–418.5]	0.737		395.2	[369.1–421.3]	395.0	[363.7–426.3]	0.993	
Potato (g)	23.4	[17.9–28.8]	28.5	[23.4–33.7]	0.181		22.1	[13–31.1]	27.0	[22.8–31.1]	0.337		26.2	[20.6–31.8]	26.0	[21.0–31.1]	0.975		25.8	[20.8–30.8]	26.6	[20.6–32.6]	0.844	
Vegetables (g)	204.9	[181.2–228.6]	207.1	[184.9–229.3]	0.896		211.5	[172.5–250.5]	204.9	[187.2–222.7]	0.765		206.5	[182.5–230.5]	205.8	[184.1–227.4]	0.965		196.6	[175.3–217.9]	219.5	[193.9–245]	0.187	
Green–yellow vegetables (g)	75.1	[65.8–84.4]	72.5	[63.8–81.2]	0.688		75.1	[59.8–90.4]	73.5	[66.5–80.4]	0.848		75.6	[66.1–85.0]	72.3	[63.8–80.8]	0.610		71.8	[63.4–80.2]	76.5	[66.5–86.6]	0.484	
Other vegetables (g)	129.8	[113.6–145.9]	134.6	[119.5–149.7]	0.673		136.4	[109.8–162.9]	131.5	[119.4–143.6]	0.743		130.9	[114.6–147.3]	133.5	[118.7–148.3]	0.819		124.8	[110.4–139.3]	142.9	[125.6–160.3]	0.125	
Seaweeds (g)	4.2	[3.3–5.0]	5.1	[4.4–5.9]	0.102		4.2	[2.8–5.6]	4.8	[4.2–5.4]	0.479		4.0	[3.1–4.8]	5.3	[4.5–6.0]	0.027	*	4.3	[3.6–5.1]	5.2	[4.3–6.1]	0.140	
Soybeans/Soy products (g)	54.3	[46.6–62.0]	61.6	[54.4–68.8]	0.175		61.5	[48.8–74.3]	57.5	[51.7–63.3]	0.571		58.1	[50.3–65.9]	58.3	[51.2–65.4]	0.968		60.0	[53.1–67]	55.6	[47.2–64]	0.435	
Fish/Processed fish (g)	68.1	[58.4–77.7]	79.9	[70.9–88.9]	0.080		62.7	[46.9–78.6]	76.9	[69.7–84.1]	0.114		66.6	[56.9–76.3]	80.7	[72.0–89.5]	0.034	*	73.5	[64.7–82.2]	75.6	[65.1–86.2]	0.760	
Meat/Processed meat (g)	78.0	[67.7–88.2]	71.6	[62.0–81.2]	0.377		68.6	[51.7–85.5]	75.9	[68.2–83.6]	0.444		73.6	[63.2–84.0]	75.3	[65.9–84.7]	0.812		74.7	[65.4–84]	74.4	[63.2–85.5]	0.965	
Eggs (g)	29.4	[25.3–33.4]	32.1	[28.3–35.9]	0.347		31.2	[24.5–37.9]	30.7	[27.7–33.8]	0.893		31.0	[26.9–35.1]	30.6	[26.9–34.4]	0.891		31.8	[28.1–35.5]	29.4	[25–33.8]	0.425	
Milk/Dairy products (g)	119.1	[98.5–139.7]	126.1	[106.8–145.4]	0.628		122.4	[88.4–156.3]	122.9	[107.5–138.4]	0.977		130.6	[109.8–151.5]	116.4	[97.6–135.3]	0.322		127.3	[108.7–145.9]	116.6	[94.2–138.9]	0.480	
Fruits (g)	82.8	[69–96.6]	81.0	[68.1–94.0]	0.855		86.9	[64.2–109.7]	80.8	[70.4–91.1]	0.629		75.8	[61.9–89.7]	86.8	[74.3–99.4]	0.250		71.7	[59.5–84]	96.2	[81.5–110.9]	0.015	*
Sweets (g)	59.9	[51.7–68.0]	50.8	[43.2–58.4]	0.115		60.7	[47.2–74.2]	53.8	[47.7–60.0]	0.366		55.3	[46.9–63.6]	54.9	[47.4–62.4]	0.950		56.1	[48.7–63.5]	53.5	[44.6–62.4]	0.664	
Alcoholic beverages (g)	116.1	[85.2–147.1]	105.1	[76.2–134.1]	0.612		124.1	[73.2–175.0]	107.3	[84.1–130.4]	0.557		103.0	[71.7–134.3]	116.2	[87.9–144.4]	0.542		120.4	[92.5–148.3]	95.9	[62.4–129.3]	0.280	
Other beverages (g)	76.6	[53.6–99.6]	53.0	[31.5–74.5]	0.145		83.2	[45.2–121.2]	59.9	[42.6–77.2]	0.276		82.6	[59.5–105.7]	48.8	[27.9–69.7]	0.035	*	62.4	[41.5–83.3]	66.3	[41.2–91.4]	0.821	
Sugar (g)	6.3	[5.2–7.4]	7.2	[6.2–8.3]	0.245		6.5	[4.6–8.4]	6.9	[6.0–7.7]	0.712		6.1	[4.9–7.2]	7.4	[6.4–8.4]	0.089		6.6	[5.6–7.7]	7.0	[5.8–8.3]	0.658	
Nuts (g)	2.2	[1.5–2.9]	2.4	[1.7–3.0]	0.780		2.2	[1.0–3.4]	2.3	[1.8–2.9]	0.813		2.2	[1.5–2.9]	2.4	[1.7–3.1]	0.685		2.1	[1.5–2.8]	2.6	[1.8–3.3]	0.400	
Oil (g)	11.9	[10.3–13.4]	12.1	[10.6–13.5]	0.846		11.5	[9.0–14.0]	12.1	[10.9–13.2]	0.694		11.6	[10–13.1]	12.3	[10.9–13.7]	0.484		12.0	[10.6–13.4]	11.9	[10.2–13.6]	0.919	
Seasonings (g)	23.7	[21.2–26.2]	26.6	[24.3–29.0]	0.097		22.8	[18.7–27.0]	25.8	[23.9–27.6]	0.210		23.9	[21.4–26.5]	26.3	[24–28.6]	0.174		25.7	[23.4–28]	24.6	[21.8–27.3]	0.545	

* *p* < 0.05. Energy intake was adjusted for age, sex, duration of diabetes, BMI, HbA1c, using OHA, and using insulin. Nutrients and food group intakes were adjusted for age, sex, duration of diabetes, BMI, HbA1c, using OHA, using insulin, and energy intake per day. BMI, body mass index; OHA, oral hypoglycemic agent.

**Table 4:** Dietary intake and response to the Diabetes Family Behavior Checklist scale for medical nutritional therapy in women with type 2 diabetes (*N* = 120).

																			Eat Foods that are not Part of					
	Praise You for Following Your Diet						Eat at the Same Time that You Do						Nag You about Following Your Diet						Your Diabetic Diet					
	None		≥ Once a Month				None		≥ Once a Month				None		≥ Once a Month				None		≥ Once a Month			
	Mean	[95%CI]	Mean	[95%CI]	*p*		Mean	[95%CI]	Mean	[95%CI]	*p*		Mean	[95%CI]	Mean	[95%CI]	*p*		Mean	[95%CI]	Mean	[95%CI]	*p*	
No. participants	76		44				20		100				85		35				60		60			
Energy (kcal)	1714	[1618–1810]	1647	[1520–1774]	0.416		1707	[1522–1893]	1686	[1604–1769]	0.836		1655	[1566–1745]	1773	[1632–1913]	0.169		1700	[1591–1809]	1680	[571–1789]	0.801	
Protein (g)	63.1	[61.3–65.0]	64.9	[62.4–67.3]	0.278		64.5	[61–68.1]	63.6	[62.0–65.2]	0.641		63.9	[62.1–65.6]	63.5	[60.8–66.3]	0.847		62.7	[60.6–64.8]	64.9	[62.8–66.9]	0.153	
Protein (% Energy)	14.8	[14.4–15.3]	15.4	[14.8–16.0]	0.124		15.2	[14.4–16.1]	15.0	[14.6–15.4]	0.653		15.0	[14.6–15.5]	15.1	[14.4–15.8]	0.930		14.7	[14.2–15.2]	15.4	[14.9–15.9]	0.054	
Fat (g)	56.3	[54.4–58.2]	57.6	[55.0–60.2]	0.431		56.0	[52.3–59.8]	56.9	[55.3–58.6]	0.659		56.9	[55.1–58.7]	56.5	[53.6–59.4]	0.818		56.1	[53.9–58.3]	57.5	[55.2–59.7]	0.409	
Fat (% Energy)	29.2	[28.1–30.3]	30.7	[29.3–32.2]	0.110		28.9	[26.8–31.0]	29.9	[29–30.9]	0.372		29.7	[28.7–30.8]	29.8	[28.2–31.5]	0.894		29.1	[27.9–30.4]	30.4	[29.1–31.6]	0.177	
Carbohydrate (g)	226.3	[221.1–231.5]	222.2	[215.3–229.1]	0.365		225.8	[215.7–235.8]	224.6	[220.1–229.1]	0.833		224.7	[219.8–229.6]	224.9	[217.2–232.7]	0.967		227.0	[221.1–232.9]	222.6	[216.7–228.5]	0.307	
Carbohydrate (% Energy)	56.0	[54.6–57.3]	53.9	[52–55.7]	0.083		55.9	[53.2–58.6]	55.1	[53.8–56.3]	0.584		55.2	[53.9–56.6]	55.1	[53.0–57.2]	0.907		56.2	[54.6–57.8]	54.2	[52.6–55.8]	0.090	
Fiber (g)	13.8	[13.1–14.5]	14.0	[13.0–15.0]	0.757		14.0	[12.6–15.4]	13.8	[13.2–14.5]	0.862		14.1	[13.5–14.8]	13.2	[12.1–14.3]	0.164		13.4	[12.6–14.2]	14.3	[13.5–15.2]	0.134	
Salt (g)	8.2	[7.7–8.6]	8.5	[7.9–9.1]	0.425		7.7	[6.9–8.6]	8.4	[8.0–8.8]	0.161		8.2	[7.8–8.6]	8.5	[7.9–9.2]	0.422		8.1	[7.6–8.6]	8.4	[7.9–8.9]	0.385	
Potassium (mg)	2274	[2172–2375]	2352	[2217–2487]	0.371		2290	[2093–2487]	2305	[2217–2393]	0.892		2318	[2222–2413]	2265	[2114–2417]	0.570		2243	[2128–2358]	2362	[2247–2477]	0.158	
Grain (g)	336.6	[317.7–355.4]	330.3	[305.2–355.3]	0.695		330.6	[294.2–367.0]	335.0	[318.8–351.2]	0.826		332.1	[314.4–349.8]	339.6	[311.6–367.5]	0.658		335.9	[314.5–357.3]	332.7	[311.2–354.1]	0.838	
Potato (g)	33.6	[27.1–40.0]	35.8	[27.3–44.4]	0.683		38.8	[26.4–51.2]	33.5	[28.0–39.1]	0.444		31.8	[25.8–37.8]	40.8	[31.4–50.3]	0.116		30.2	[23–37.5]	38.6	[31.3–45.8]	0.120	
Vegetables (g)	261.4	[235.7–287.0]	275.6	[241.5–309.7]	0.518		259.3	[209.7–308.9]	268.0	[246.0–290.1]	0.751		276.9	[253–300.8]	241.5	[203.8–279.2]	0.124		252.5	[223.5–281.4]	280.7	[251.7–309.7]	0.185	
Green–yellow vegetables (g)	91.9	[81.2–102.7]	97.5	[83.2–111.8]	0.550		89.9	[69–110.7]	94.8	[85.5–104.1]	0.668		96.8	[86.7–106.9]	87.0	[71.1–103.0]	0.312		88.5	[76.3–100.6]	99.5	[87.3–111.7]	0.217	
Other vegetables (g)	169.4	[152.9–185.9]	178.1	[156.2–200.0]	0.538		169.4	[137.6–201.3]	173.2	[159.1–187.4]	0.830		180.1	[164.8–195.3]	154.5	[130.4–178.6]	0.083		164.0	[145.4–182.6]	181.2	[162.6–199.8]	0.209	
Seaweeds (g)	5.8	[4.9–6.7]	6.0	[4.9–7.2]	0.734		6.4	[4.7–8.1]	5.8	[5.0–6.5]	0.493		6.0	[5.2–6.8]	5.6	[4.2–6.9]	0.557		5.4	[4.5–6.4]	6.3	[5.3–7.3]	0.237	
Soybeans/Soy products (g)	69.7	[60.3–79.2]	71.6	[59–84.1]	0.821		76.2	[58–94.4]	69.2	[61.2–77.3]	0.491		73.1	[64.3–81.9]	63.8	[49.9–77.7]	0.271		64.8	[54.2–75.4]	76.0	[65.4–86.6]	0.150	
Fish/Processed fish (g)	70.8	[63.2–78.5]	72.2	[62–82.3]	0.838		75.0	[60.3–89.7]	70.6	[64.0–77.1]	0.588		69.8	[62.6–76.9]	75.2	[63.9–86.5]	0.429		71.6	[62.9–80.3]	71.1	[62.4–79.7]	0.934	
Meat/Processed meat (g)	67.6	[59.1–76.1]	69.0	[57.7–80.3]	0.851		65.6	[49.2–82]	68.6	[61.3–75.9]	0.737		67.0	[59.0–74.9]	70.9	[58.3–83.5]	0.603		64.5	[54.9–74.1]	71.7	[62.1–81.3]	0.306	
Eggs (g)	23.3	[19.9–26.6]	23.3	[18.9–27.7]	0.996		27.3	[21.0–33.7]	22.5	[19.6–25.3]	0.170		24.2	[21.1–27.3]	21.1	[16.2–26.0]	0.300		21.8	[18–25.5]	24.8	[21–28.5]	0.281	
Milk/Dairy products (g)	125.7	[107.8–143.5]	144.6	[120.9–168.4]	0.217		119.0	[84.4–153.6]	135.4	[120–150.8]	0.395		131.4	[114.5–148.3]	135.6	[109–162.3]	0.792		136.5	[116.1–156.9]	128.7	[108.3–149.1]	0.603	
Fruits (g)	100.7	[87.4–114.0]	94.2	[76.5–111.9]	0.571		98.2	[72.4–123.9]	98.3	[86.9–109.8]	0.991		94.5	[82.0–106.9]	107.6	[87.9–127.3]	0.272		105.1	[90–120.1]	91.5	[76.5–106.6]	0.220	
Sweets (g)	60.2	[52.1–68.3]	51.2	[40.4–62.0]	0.195		61.8	[46.1–77.5]	55.9	[48.9–62.9]	0.501		60.1	[52.5–67.7]	49.2	[37.2–61.2]	0.135		61.7	[52.5–70.8]	52.2	[43–61.4]	0.161	
Alcoholic beverages (g)	21.7	[11.6–31.7]	16.6	[3.3–30.0]	0.556		18.6	[–0.9–38]	20.1	[11.4–28.7]	0.889		18.4	[9.0–27.8]	23.3	[8.4–38.2]	0.589		22.5	[11.1–33.9]	17.2	[5.7–28.6]	0.524	
Other beverages (g)	21.3	[7.3–35.2]	35.4	[16.9–54.0]	0.237		19.3	[–7.8–46.3]	27.9	[15.8–39.9]	0.567		23.8	[10.7–37.0]	32.8	[12.1–53.6]	0.474		25.3	[9.4–41.3]	27.6	[11.6–43.5]	0.847	
Sugar (g)	8.0	[7.0–9.1]	8.0	[6.6–9.3]	0.941		7.4	[5.5–9.4]	8.1	[7.2–9.0]	0.547		7.4	[6.4–8.3]	9.6	[8.1–11.1]	0.015	*	7.5	[6.3–8.6]	8.5	[7.4–9.7]	0.212	
Nuts (g)	3.6	[2.4–4.8]	3.1	[1.5–4.7]	0.620		2.3	[0.1–4.6]	3.7	[2.6–4.7]	0.299		3.6	[2.5–4.7]	3.0	[1.2–4.7]	0.523		3.2	[1.8–4.5]	3.7	[2.4–5]	0.583	
Oil (g)	10.6	[9.0–12.2]	12.2	[10–14.3]	0.259		8.6	[5.4–11.7]	11.7	[10.3–13.1]	0.072		10.7	[9.1–12.2]	12.4	[10.0–14.8]	0.245		11.3	[9.4–13.1]	11.0	[9.2–12.9]	0.856	
Seasonings (g)	18.6	[16.6–20.6]	20.5	[17.8–23.2]	0.271		17.6	[13.7–21.5]	19.7	[17.9–21.4]	0.349		19.3	[17.4–21.2]	19.3	[16.3–22.4]	0.990		18.8	[16.4–21.1]	19.9	[17.6–22.2]	0.507	

* *p* < 0.05. Energy intake was adjusted for age, sex, duration of diabetes, BMI, HbA1c, using OHA, and using insulin. Nutrients and food group intakes were adjusted for age, sex, duration of diabetes, BMI, HbA1c, using OHA, using insulin, and energy intake per day. BMI, body mass index; OHA, oral hypoglycemic agent.
